# Rutin Increases Muscle Mitochondrial Biogenesis with AMPK Activation in High-Fat Diet-Induced Obese Rats

**DOI:** 10.3390/nu7095385

**Published:** 2015-09-22

**Authors:** Sangjin Seo, Mak-Soon Lee, Eugene Chang, Yoonjin Shin, Soojung Oh, In-Hwan Kim, Yangha Kim

**Affiliations:** 1 Department of Nutritional Science and Food Management, Ewha Womans University, Seoul 120-750, Korea; E-Mails: sjseo27@naver.com (S.J.S.); troph@hanmail.net (M.S.L.); eugenics77@hotmail.com (E.C.); yjin19@hotmail.com (Y.S.); ohsjmay@naver.com (S.O.); 2 Department of Food and Nutrition, Korea University, Seoul 136-703, Korea; E-Mail: k610in@korea.ac.kr

**Keywords:** rutin, obesity, skeletal muscle, mitochondria, AMPK activity

## Abstract

Decreased mitochondrial number and dysfunction in skeletal muscle are associated with obesity and the progression of obesity-associated metabolic disorders. The specific aim of the current study was to investigate the effects of rutin on mitochondrial biogenesis in skeletal muscle of high-fat diet-induced obese rats. Supplementation with rutin reduced body weight and adipose tissue mass, despite equivalent energy intake (*p* < 0.05). Rutin significantly increased mitochondrial size and mitochondrial DNA (mtDNA) content as well as gene expression related to mitochondrial biogenesis, such as peroxisome proliferator-activated receptor γ coactivator-1α (PGC-1α), nuclear respiratory factor-1 (NRF-1), transcription factor A (Tfam), and nicotinamide adenine dinucleotide (NAD)-dependent deacetylase, sirtulin1 (SIRT1) in skeletal muscle (*p* < 0.05). Moreover, rutin consumption increased muscle adenosine monophosphate-activated protein kinase (AMPK) activity by 40% (*p* < 0.05). Taken together, these results suggested at least partial involvement of muscle mitochondria and AMPK activation in the rutin-mediated beneficial effect on obesity.

## 1. Introduction

The rapidly increased prevalence of obesity has become a worldwide health epidemic due to its strong association with metabolic disorders, including type 2 diabetes, dyslipidemia, hypertension, and heart disease [1,2]. Skeletal muscle from obese humans shows increased intramuscular triglyceride content and decreased lipid oxidation [3,4]. In addition, decreased activities of enzymes indicative of muscle mitochondrial content and mitochondrial number in obese human skeletal muscle have also been reported [4,5,6]. Given the close association between obesity and skeletal muscle, the regulation of obesity-induced mitochondrial changes in skeletal muscle may be possible targets for the prevention and/or treatment of obesity and its associated comorbidities.

Mitochondrial dysfunction, including mitochondrial loss and decreased functional capacity of mitochondria, is associated with several transcriptional regulators and enzyme activities in obese skeletal muscle. Adenosine monophosphate-activated protein kinase (AMPK) stimulates mitochondrial biogenesis and β-oxidation by regulating a transcriptional regulation factor, peroxisome proliferator-activated receptor γ coactivator-1α (PGC-1α) [7,8,9]. PGC-1α directly increases the synthesis of nuclear respiratory factors (NRF1 and NRF2) and mitochondrial transcription factor A (Tfam), all of which increase mitochondrial DNA (mtDNA), an indicator of mitochondrial biogenesis and mitochondrial function [10,11]. In studies that investigated the association between mitochondrial biogenesis and obesity, genetic variation in the mtDNA demonstrates a close association with the severity of obesity [12,13]. A nicotinamide adenine dinucleotide (NAD)-dependent deacetylase, sirtulin1 (SIRT1) also interacts with PGC-1α, which plays a critical role in apoptosis, energy homeostasis, longevity, and mitochondrial function [14]. Another transcriptional candidate for mitochondrial function is carnitine palmitoyltransferase 1 (CPT1). CPT1 is a mitochondrial transmembrane enzyme that regulates the entry of long-chain fatty acids into mitochondria for fatty acid oxidation [15]. In obese human skeletal muscle, CPT1 activity and mitochondrial content are decreased, which in turn contribute to reduced fatty acid oxidation [4].

Rutin (rutoside, quercetin-3-*O*-rutinoside and sophorin) is a flavonol glycoside composed of quercetin and disaccharide rutinose and is present in many plants, including buckwheat [16]. *In vivo* and *in vitro* studies showed the anti-oxidant, anti-inflammatory, anti-hypertensive, and anti-platelet properties of rutin [17,18] by modulating oxidative stress, inflammation, lipogenesis, and glucose and lipid metabolism in liver and adipose tissue [19,20,21,22]. However, the favorable effect of rutin on obesity in relation to muscle mitochondrial changes has never been investigated. Thus, the purpose of our study was to determine the effect of rutin on obesity-induced mitochondrial loss and decreased functional capacity in skeletal muscle from high-fat diet-induced obese rats.

## 2. Experimental Section

### 2.1. Reagents

Trizol reagent and Moloney murine leukemia virus (M-MLV) reverse transcriptase were obtained from Invitrogen (Carlsbad, CA, USA). A universal SYBR Green polymerase chain reaction (PCR) Master Mix and Puregene DNA isolation kit were obtained from Qiagen (Valencia, CA, USA). Kits for the analysis of aspartate aminotransferase (AST), alanine aminotransferase (ALT), total cholesterol (TC), triglyceride (TG), and high density lipoprotein (HDL)-cholesterol were purchased from Asan Pharmaceutical Co. (Seoul, Korea). The AMPK Kinase Assay kit was obtained from MBL International Co. (Woburn, MA, USA). The BCA protein assay kit was purchased from Thermo Scientific (Waltham, MA, USA). Zoletil was provided by Virbac Laboratories (Carros, France). Rompun was supplied by Bayer Korea (Ansan, Korea). All other reagents including rutin were obtained from Sigma-Aldrich Inc. (St. Louis, MO, USA).

### 2.2. Animals and Experimental Design

Experimental protocols were approved by the Animal Experimentation Ethics Committee of Ewha Womans University in Seoul, Korea for the care and use of laboratory animals (permission number: 2012-02-080). A total of 24 male Sprague-Dawley rats (3 weeks old) were obtained from Daehan Experimental Animals (Eumseong, Korea), individually housed in stainless steel wire-mesh cages in a room maintained at 22 ± 2 °C with a 12-h light/dark cycle (light period: 6 am to 6 pm), and fed laboratory chow and water ad libitum for 1 week to stabilize their metabolic condition. After 1 week of acclimation, rats were randomly divided into groups (*n* = 8/group) and fed a normal diet (NOR), a high-fat diet (HFD) or a 0.1% (wt:wt) rutin-supplemented high-fat diet (HFD + Rutin) for 12 weeks. NOR was a commercial diet (Harlan 2018s; Harlan, Indianapolis, IN, USA) containing 18% crude protein, 6% fat, 44% carbohydrate, 18% fiber, and 5% ash. HFD (45% of energy) consisted of 23% fat, 17% casein, 12% sucrose, 20% starch, 15% dextrose, 6% cellulose, 4.3% minerals, and 1.2% vitamins (wt:wt), based on a modification of the AIN-93 diet [23]. Rutin was added to HFD. Body weight and food intake were monitored twice a week.

### 2.3. Sample Collection

At the end of 12 weeks, rats fasted overnight and were anesthetized with Zoletil:Rompun (4:1) at a dose of 0.1 mL/80 g body weight. Blood was collected by cardiac puncture, centrifuged at 1500× *g* for 20 min at 4 °C to obtain serum, and stored at −20 °C until analysis. Liver, white adipose tissue (epididymal and retroperitoneal), and skeletal muscle were dissected, immediately frozen in liquid nitrogen, and stored at −70°C until further analysis.

### 2.4. Blood Biochemical Measurements

Serum concentrations of AST, ALT, TC, TG and HDL-cholesterol were determined by enzymatic colorimetric methods using commercial kits (Asan Pharmaceutical Co., Ltd., Seoul, Korea). Low-density lipoprotein (LDL)-cholesterol was calculated using the Friedewald equation [24], and the atherogenic index (AI) was calculated using the Rosenfeld formula [25].

LDL-cholesterol = TC – HDL-cholesterol – (TG/5)(1)

AI = (TC – HDL-cholesterol)/HDL-cholesterol(2)

### 2.5. Hepatic and Fecal Lipids Analyses

Hepatic and fecal lipids were extracted using the method described by Bligh and Dyer [26]. Briefly, 500 mg of tissues or feces was homogenized in 1.5 mL of 0.9% saline and 7.5 mL of methanol:chloroform (2:1, v:v). After the addition of 2.5 mL of chloroform, the mixture was shaken horizontally for 10 min and centrifuged at 2000× *g* for 10 min. The lower chloroform phase was collected into a fresh tube and subsequently dried and weighed. TC and TG concentrations were determined by enzymatic colorimetric methods using commercial kits as described above.

### 2.6. Histological Analysis

Dissected epididymal adipose tissue samples were fixed in 10% (v/v) formalin for 24 h. Tissues were then embedded in paraffin, sliced into 5-μm-thick sections, and stained with hematoxylin-eosin (H&E). H&E-stained sections were observed under a microscope (Olympus, Tokyo, Japan) and digital images were captured at 200× magnification.

### 2.7. Electron Microscopic Analysis

Skeletal muscle was prefixed with 2% glutaraldehyde plus 2% paraformaldehyde in 0.1 M phosphate buffer. Glutaraldehyde-fixed samples were treated with 2% osmium tetroxide, dehydrated, and embedded in epoxy resin. Selected 1-μm-thick sections were stained with toluidine blue and then cut into approximately 60- to 70-μm ultra-thin sections using an Ultramicrotome (Richert-Jung, Buffalo, NY, USA) using a diamond knife. Thin sections were stained with 1%–2% aqueous uranyl acetate, followed by 1% lead citrate. Stained sections were examined using an H-7650 transmission electron microscope (Hitachi, Japan) at the accelerating voltage of 80 kV.

### 2.8. Real-time Quantitative Reverse-transcription Polymerase Chain Reaction (qRT-PCR)

The isolation of RNA from epididymal adipose tissue and skeletal muscle was performed using Trizol reagent. Complementary DNA (cDNA) was synthesized from 4 μg of total RNA using a M-MLV Reverse Transcriptase Kit. Primers used are shown in Table 1. Real-time quantitative reverse-transcription polymerase chain reaction (qRT-PCR) was carried out using Universal SYBR Green PCR Master Mix on a fluorometric thermal cycler (Rotor-GeneTM 2000; Corbett Research, Mortlake, NSW, Australia). Data were analyzed using the ΔΔCt method for relative quantification [27]. The expression of each target was normalized to the average of β-actin as a control and expressed as the fold change related to the HFD group.

**Table 1:** Primers used for quantitative real-time polymerase chain reaction (PCR).

Name	GeneBank No.	Primer sequence (5’-3’)	Amplicon Size (bp)
aP2	NM_053365	F: TCACCCCAGATGACAGGAAA	140
		R: CATGACACATTCCACCACCA	
β-actin	NM_031144	F: GGCACCACACTTTCTACAAT	123
		R: AGGTCTCAAACATGATCTGG	
CPT-1	NM_031559	F: TCGGCAGACCTATTTTGCAC	143
		R: ATTTGGCGTAGCTGTCGATG	
		R: ATGGCTTGAGGATCTGGGAG	
NRF1	NM_001100708	F: CTGTGGCTGATGGAGAGGTG	189
		R: CACTGTTAAGGGCCATGGTG	
PGC-1α	NM_031347	F: GCACCAGAAAACAGCTCCAA	130
		R: TTACTGAAGTTGCCATCCCG	
SIRT1	XM_003751934.1	F: GTTCTGACTGGAGCTGGGGT	119
		R: ATGGCTTGAGGATCTGGGAG	
SREBP-1c	AF286470	F: AGGAGGCCATCTTGTTGCTT	134
		R: GTTTTGACCCTTAGGGCAGC	
PPAR-γ	NM_001145366	F: TGTGGGGATAAAGCATCAGC	175
		R: CAAGGCACTTCTGAAACCGA	
Tfam	NM_031326	F: TGGGCTTAGAGAAGGAAGCC	107
		R: TGCTGACCGAGGTCTTTTTG	

aP2, fatty acid-binding protein 2; CPT-1, carnitine palmitoyltransferase 1; NRF1, nuclear respiratory factor 1; PGC-1α, peroxisome proliferative activated receptor gamma coactivator 1 alpha; PPAR-γ, peroxisome proliferator-activated receptor-γ; SIRT1, sirtuin 1; SREBP-1c, sterol regulatory element-binding protein-1c; Tfam, mitochondrial transcription factor A.

### 2.9. Mitochondrial DNA (mtDNA) Content Analysis

Total DNA was extracted from muscle using a Puregene DNA isolation kit. The mtDNA content was calculated using real-time quantitative PCR by measuring the mitochondrial gene (Cox1, subunit 1 of cytochrome oxidase) *vs.* nuclear gene (GAPDH, glyceraldehyde 3-phosphate dehydrogenase).

### 2.10. AMPK Activity Assay

AMPK activity was evaluated using an AMPK Kinase Assay kit as described previously [28]. Using a semi-quantitative method, AMPK activity was detected by measuring the phosphorylation of Ser789 on IRS-1 with anti-mouse phospho-Ser789 IRS-1 monoclonal antibody and peroxidase-coupled anti-mouse IgG, which catalyzes the conversion of the chromogenic substrate tetramethylbenzidine. Protein was determined using a BCA protein assay kit. AMPK activity was normalized to protein concentration and expressed as the fold change compared to the HFD group.

### 2.11. Statistical Analysis

Data are expressed as the mean ± standard error of the mean (SEM). Differences among groups were determined by Student’s *t*-test for the comparison of two groups or one-way analysis of variance (ANOVA) following Tukey’s multiple comparison using SPSS software (version 17; IBM Corporation, Armonk, NY, USA). Statistical significance was defined as *p* < 0.05.

## 3. Results

### 3.1. Effect of Rutin Supplementation on Body Weight, Energy Intake, and Fat Accumulation

**Figure 1:**
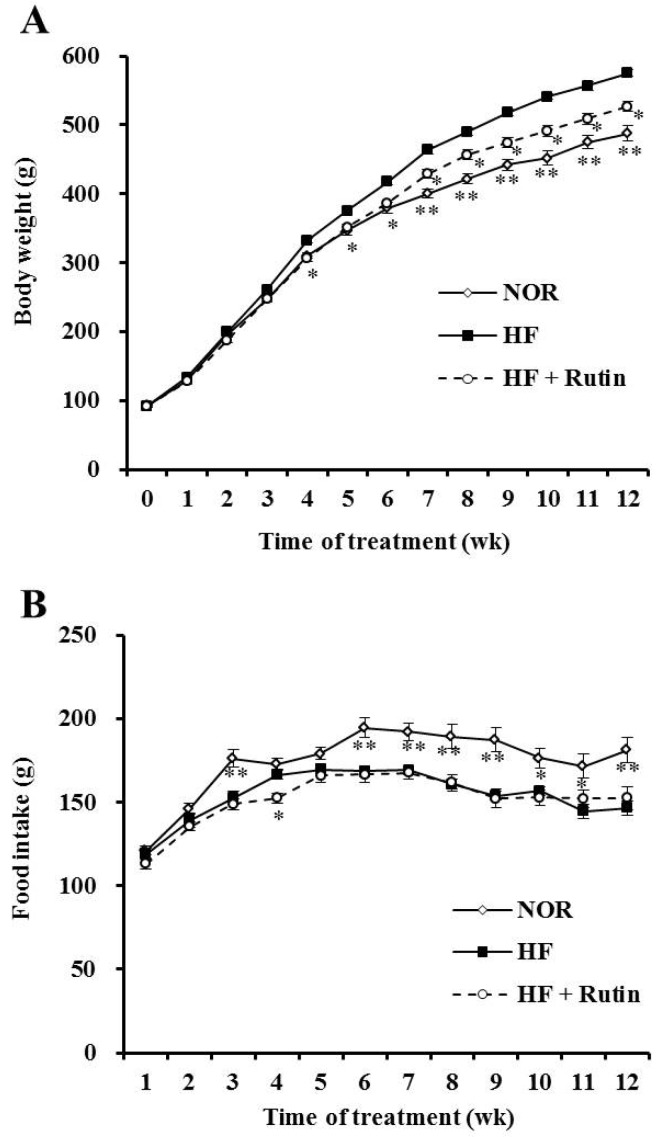

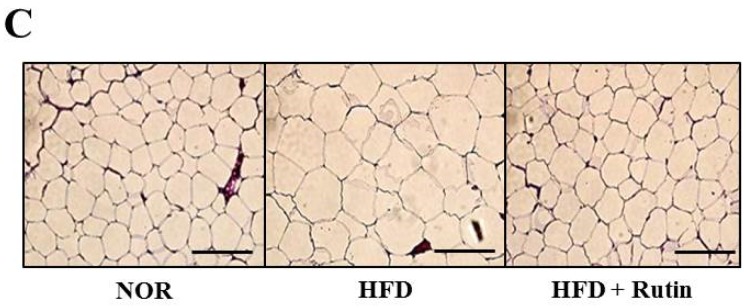
Effect of rutin on diet-induced obesity. Changes in body weight (**A**) and food intake (**B**). Representative histological sections (**C**) of epididymal adipose tissue (hematoxylin and eosin stain, scale bar = 50 μm). The data are expressed as the mean ± standard error of the mean (SEM) (*n* = 8 per group). * *p* < 0.05; ** *p* < 0.01 compared to the high-fat diet (HFD; 45% of energy) group. NOR, normal diet; HFD, high-fat diet; HFD + Rutin, rutin-supplemented high-fat diet.

At the beginning of the experiment, the initial body weight was not significantly different among groups. After 12 weeks of rutin consumption, final body weight was significantly decreased by 8.5% compared to the HFD group (*p* < 0.05) (Figure 1A and Table 2). The HFD + Rutin group showed a significantly lower total weight of epididymal and retroperitoneal adipose tissues by 24% than total adipose tissue weight of HFD group (*p* < 0.05) (Table 2). As shown in Figure 1C, the size of epididymal adipocytes was smaller in the HFD + Rutin group than in the HFD group. The food intake and energy efficiency ratio were not significantly different between HFD and HFD + Rutin, which indicate that the beneficial effects of rutin on body weight and the mass of white adipose tissue were not caused by reduced energy consumption (Figure 1B and Table 2).

**Table 2:** Effect of rutin on body weight, food intake, and tissue weight.

Variables	NOR	HFD	HFD + Rutin
Initial body weight (g)	92.36 ± 1.41	91.10 ± 1.76	92.12 ± 1.68
Final body weight (g)	487.40 ± 12.59 ^c^	573.64 ± 7.22 ^a^	528.54 ± 9.05 ^b^
Food intake (g/day)	24.97 ± 0.57 ^a^	21.96 ± 0.30 ^b^	21.63 ± 0.38 ^b^
Total food intake (kg/12 weeks)	2.02 ± 0.05 ^a^	1.78 ± 0.02 ^b^	1.75 ± 0.03 ^b^
Food efficiency (g gain/g consumed)	0.19 ± 0.002 ^c^	0.27 ± 0.003 ^a^	0.25 ± 0.006 ^b^
Energy intake (kcal/day)	77.42 ± 1.77 ^b^	101.99 ± 1.40 ^a^	100.45 ± 1.79 ^a^
Total energy intake (kcal/12 weeks)	6271 ± 144 ^b^	8261 ± 113 ^a^	8137 ± 145 ^a^
Energy efficiency (g gain/kcal consumed)	0.06 ± 0.001 ^a^	0.06 ± 0.001 ^a^	0.05 ± 0.001 ^b^
Liver weight (g/100 g body weight)	2.65 ± 0.06	2.44 ± 0.03	2.54 ± 0.09
Adipose tissue weight (g/100 g body weight)			
Epididymal	2.30 ± 0.18 ^b^	3.84 ± 0.16 ^a^	3.28 ± 0.18 ^a^
Retroperitoneal	2.28 ± 0.16 ^b^	4.29 ± 0.19 ^a^	2.94 ± 0.26 ^b^
Total	4.58 ± 0.33 ^b^	8.14 ± 0.29 ^a^	6.22 ± 0.33 ^b^

The data are expressed as the mean ± standard error of the mean (SEM) (*n* = 8). ^a, b, c^ Different letters indicate a significant difference among groups according to Tukey’s multiple comparison test (*p* < 0.05). NOR, normal diet; HFD, high-fat diet.

### 3.2. Effect of Rutin on Serum Lipid Profiles

HFD-increased serum concentrations in TG and LDL-cholesterol showed 43% and 55% reduction in the rutin-supplemented group, respectively (*p* < 0.05). Rutin consumption significantly increased the serum HDL-cholesterol level by 62% compared to HFD group (*p* < 0.05), resulting in a significant decrease of the atherogenic index (AI) (Table 3).

**Table 3:** Effect of rutin on serum lipid profiles.

Serum Lipid Profiles	NOR	HFD	HFD + Rutin
Serum lipids (mmol/L)			
Triglyceride	1.03 ± 0.12 ^a,b^	1.25 ± 0.09 ^a^	0.71 ± 0.04 ^b^
Total cholesterol	2.32 ± 0.14 ^a,b^	2.65 ± 0.12 ^a^	2.16 ± 0.15 ^a,b^
HDL cholesterol	1.45 ± 0.21 ^b^	1.3 ± 0.13 ^b^	2.1 ± 0.32 ^a^
LDL cholesterol	0.7 ± 0.17 ^a,b^	1.09 ± 0.12 ^a^	0.49 ± 0.22 ^b^
Atherogenic index (AI)	0.91 ± 0.27 ^a,b^	1.7 ± 0.36 ^a^	0.51 ± 0.24 ^b^
AST (IU/L)	65.8 ± 4.0	69.7 ± 4.1	66.0 ± 2.5
ALT (IU/L)	8.8 ± 3.1	6.2 ± 2.4	7.2 ± 2.9

Values are shown as the mean ± standard error of the mean (SEM) (*n* = 8). ^a, b^ Different letters indicate a significant difference among groups according to Tukey’s multiple comparison test (*p* < 0.05). NOR, normal diet; HFD, high-fat diet; HDL, high-density lipoprotein; LDL, low-density lipoprotein; AST, aspartate aminotransferase; ALT, alanine aminotransferase; IU, international unit.

### 3.3. Alterations in Hepatic and Fecal Lipid Profiles by Rutin Supplementation

Twelve weeks of rutin supplementation significantly reduced the hepatic total lipid, TG, and TC by 27%, 37%, and 35%, respectively, compared to the HFD group (*p* < 0.05) (Table 4). The amount of fecal lipid in the HFD + Rutin group was significantly increased by 33% compared to the HFD group (*p* < 0.05). There was an increasing trend of fecal TG levels in the HFD + Rutin group, but there was no significance.

**Table 4:** Effect of rutin on hepatic and fecal lipid profiles.

Hepatic and Fecal Lipid Profiles	NOR	HFD	HFD + Rutin
Hepatic lipids (μmol/g)			
Total lipid	26.7 ± 1.3 ^b^	42.2 ± 1.3 ^a^	30.9 ± 1.9 ^b^
Triglyceride	3.79 ± 0.69 ^c^	11.70 ± 1.76 ^a^	7.33 ± 1.36 ^b^
Total cholesterol	1.26 ± 0.10 ^b^	2.70 ± 0.21 ^a^	1.75 ± 0.13 ^b^
Fecal lipids (μmol/g)			
Total lipid	23.9 ± 1.6 ^b^	28.5 ± 1.0 ^a,b^	38.0 ± 3.8 ^a,^*
Triglyceride	0.36 ± 0.04 ^b^	0.47 ± 0.04 ^a,b^	0.66 ± 0.09 ^a^

Values are expressed as the mean ± standard error of the mean (SEM) (*n* = 8). ^a, b, c^ Different letters indicate a significant difference among groups according to Tukey’s multiple comparison test (*p* < 0.05). Asterisks (*) indicate a significant difference from HF diet, *p* < 0.05. NOR, normal diet; HFD, high-fat diet.

### 3.4. Influence of Rutin on Liver Weight and Serum AST and ALT Activities

Liver weight was not altered by rutin supplementation (Table 2). In addition, serum levels of AST and ALT were not significantly different among groups (Table 3). The data indicated that rutin did not induce hepatic toxicity.

### 3.5. Effect of Rutin on Expression of Adipogenic Genes and AMPK Activity in Adipose Tissue

**Figure 2:**
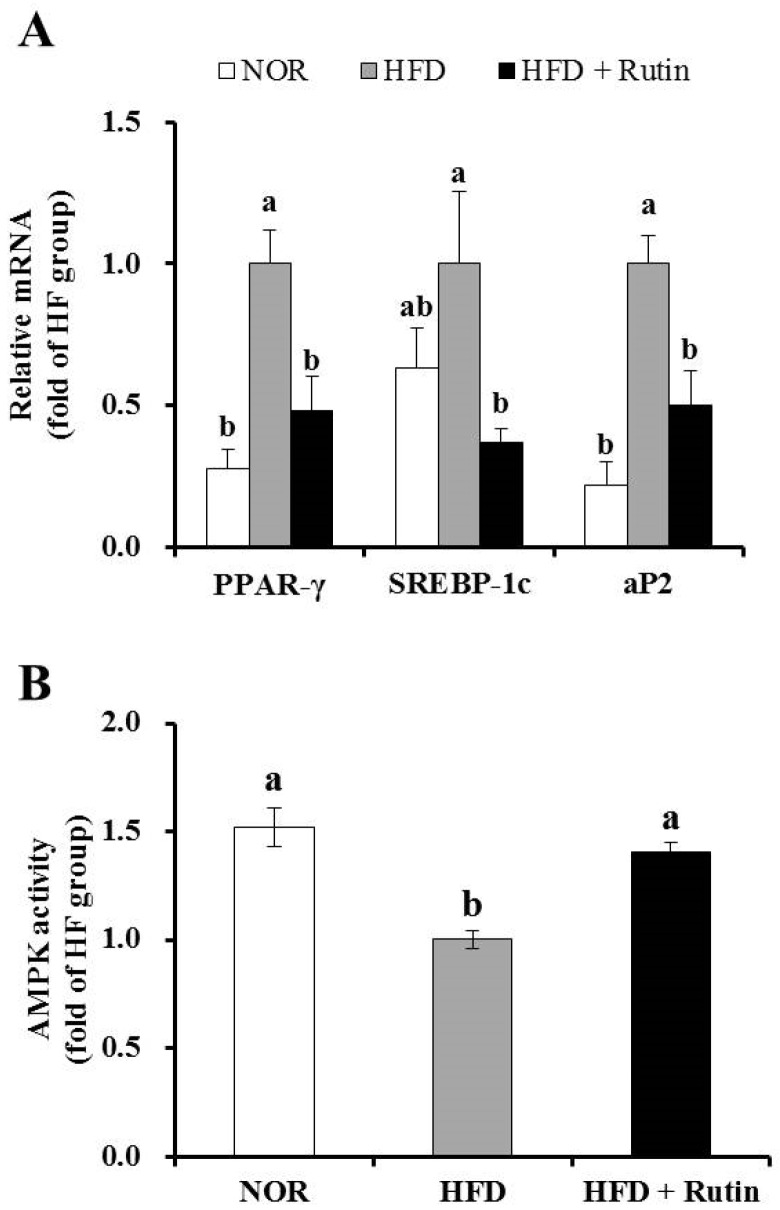
Effect of rutin on adipogenic gene expression and adenosine monophosphate-activated protein kinase (AMPK) activity in adipose tissue. The messenger RNA (mRNA) levels of peroxisome proliferator activated receptor-γ (PPAR-γ), sterol regulatory element binding protein-1c (SREBP-1c) and adipocyte protein 2 (aP2) were determined by real-time polymerase chain reaction (RT-PCR) and normalized for all samples to β-actin (**A**). AMPK activity was measured using an AMPK kinase kit and normalized to protein levels (**B**). The results are expressed as the fold change compared to the HFD group (mean ± standard errof of the mean (SEM), *n* = 8 per group). Bars with different letters (a, b) are significantly different according to Tukey’s multiple comparison test at *p* < 0.05.

The mRNA levels of adipogenic genes such as peroxisome proliferator activated receptor-γ (PPAR-γ), sterol regulatory element binding protein-1c (SREBP-1c), and adipocyte protein 2 (aP2) were significantly decreased by rutin in epididymal white adipose tissue (*p* < 0.05) (Figure 2A). AMPK activation, which regulates body fat accumulation by modulating adipogenic or fatty acid oxidation-related genes, was measured by a semi-quantitative analysis. As shown in Figure 2B, the HFD + Rutin group exhibited significantly increased AMPK activity by 50% in adipose tissue compared to the HFD group (*p* < 0.05). Therefore, the favorable effects of rutin on body weight and adipose tissue mass may be associated with a decrease of adipogenesis in adipose tissue.

### 3.6. Effect of Rutin on Mitochondrial Morphology and Content (mtDNA) in Skeletal Muscle

**Figure 3:**
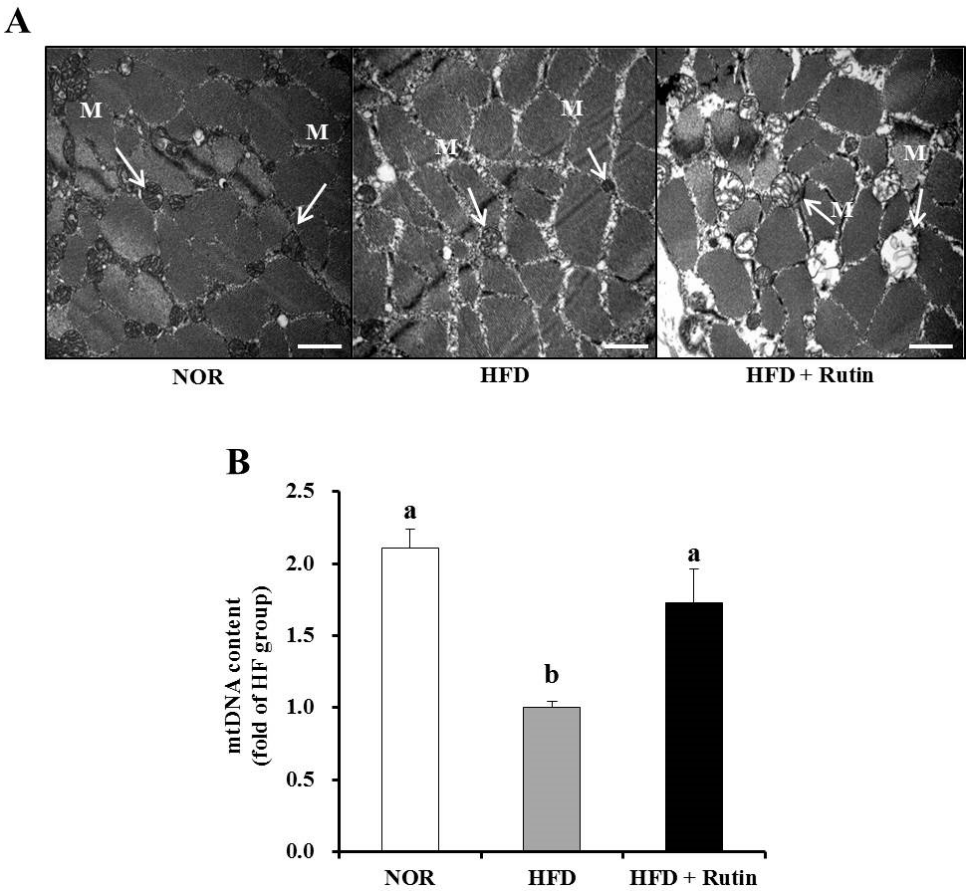
Effect of rutin on muscle mitochondrial morphology and mitochondrial DNA (mtDNA) content. Electron microscopy of muscle (magnification of 20,000; scale bars = 2 µm) (**A**). Arrows indicate the position of mitochondria (M). The mtDNA content was quantified by real-time PCR (**B**). Values are expressed as the mean ± standard error of the mean (SEM) (*n* = 8 per group). Bars with different letters (a, b) indicate significant differences compared to the HFD group according to Tukey’s multiple comparison test (*p* < 0.05).

To investigate the effect of rutin on changes in muscle mitochondria, transmission electron microscopy (TEM) was used. The HFD group showed a smaller size and number of skeletal muscle mitochondria, which was reversed by rutin administration (Figure 3A). In addition, Figure 3B showed that mtDNA content was significantly increased by rutin supplementation, which served as evidence of enlargement and increased mitochondria number (*p* < 0.05).

### 3.7. Effect of Rutin on Mitochondrial Gene Expression and AMPK Activity in Skeletal Muscle

**Figure 4:**
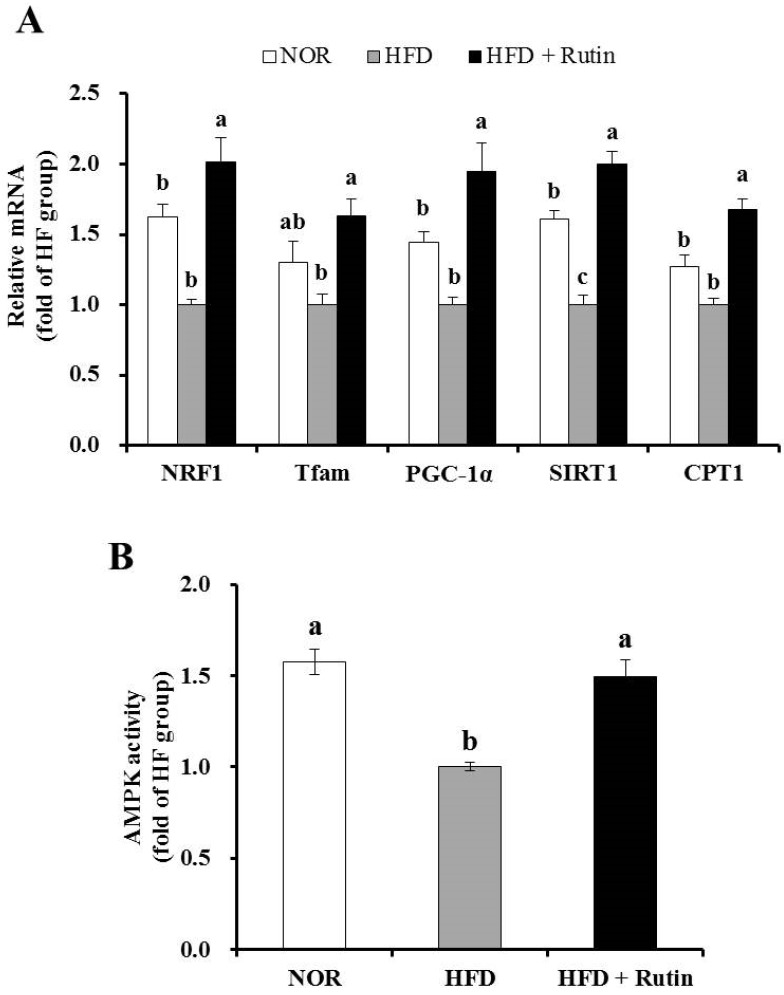
Effect of rutin on mitochondrial gene expression of genes related to mitochondrial biogenesis and function in skeletal muscle. The levels of nuclear respiratory factor 1 (NRF1), transcription factor A (Tfam), peroxisome proliferator-activated receptor γ coactivator-1α (PGC-1α), sirtulin1 (SIRT1) and arnitine palmitoyltransferase 1 (CPT1) mRNA were analyzed by real-time PCR and normalized to β-actin (**A**). adenosine monophosphate-activated protein kinase (AMPK) activity was measured using an AMPK kinase kit and normalized to protein levels (**B**). The results are expressed as the fold change compared to the HFD group (mean ± standard error of the mean (SEM), *n* = 8 per group). Bars with different letters (a, b) indicate a statistically significant difference according to Tukey’s multiple comparison test (*p* < 0.05).

Rutin administration significantly increased mRNA expression of NRF1, Tfam, PGC-1α, SIRT1, and CPT1 involved in muscle mitochondrial biogenesis and function (*p* < 0.05) (Figure 4A). Next, we determined AMPK activity, which affects muscle mitochondrial biogenesis and oxidative capacity for β-oxidation. Rutin-supplemented HFD significantly increased AMPK activity by 40% in muscle compared to the HFD group (Figure 4B). The data indicated that the beneficial effect of rutin on obesity may be due to an increase in mitochondrial biogenesis and capacity in skeletal muscle.

## 4. Discussion

Mitochondrial changes and dysfunction, including decreased mtDNA content, mitochondrial size, and functional capacity of mitochondria, manifest in the skeletal muscle of obese human subjects and rodents [4,5,6,29,30,31]. Beneficial effects of rutin supplementation on obesity have been reported [19,20,22,32]. The favorable role of rutin in glucose and lipid metabolism and its associated metabolic disorders led us to investigate the effects of rutin on obesity-induced mitochondrial changes in skeletal muscle.

In the present study, we evaluated the influence of rutin on the HFD-decreased mitochondrial content, mitochondrial gene expression, and AMPK activity, which is involved in mitochondrial biogenesis and function. Using three groups of rats fed a NOR, HFD and HFD + Rutin, we found that rutin supplementation led to significant increases in the HFD-decreased size and number of mitochondria, mtDNA and gene expression and enzyme activity related to mitochondrial biogenesis and function in skeletal muscle, together with reductions in HFD-induced weight gain and the enlargement of adipose tissue.

In the present study, rutin-fed rats showed a significant decrease in body weight without changing food intake, consistent with other previous results [21,32]. Moreover, rutin consumption decreased adipose size, adipogenic gene expression of PPAR-γ, SREBP-1c, and aP2, and AMPK activity in epididymal adipose tissue, indicating the anti-obesity property of rutin. PPAR-γ and SREBP-1c are critical transcription factors that regulate adipocyte differentiation and lipogenesis [33,34]. In addition, aP2 is highly expressed in adipose tissue and regarded as a marker of obesity, reflecting the magnitude of fat accumulation [35]. In adipocyte energy homeostasis, AMPK regulates body fat deposition by decreasing adipogenic genes such as SREBP-1c and PPAR-γ [36]. Adipogenic gene expression and adipose tissue hypertrophy were inhibited *in vitro* in 3T3-L1 cells treated with rutin and animals fed with rutin supplemented with a high-fat diet [19,20]. Therefore, our findings demonstrated that the beneficial effect of rutin on obesity was at least partially due to anti-adipogenic activity in adipose tissue.

The currently used rutin dosage was determined as described in previous studies [21,37]. A concentration of 0.1% rutin in the diet was well tolerated by rats in the present study, as demonstrated by the results showing that liver weight and serum ALT and AST levels were unaffected by rutin supplementation. In rodent animal models, HFD generally does not induce liver damage. When fed with 60% HFD, 16 weeks period of HFD significantly increases hepatic expression involved in inflammation [38]. In addition, high-fat liquid diet including 71% of energy derived from fat induces hepatic inflammation after 4 weeks [39] or 6 weeks [40]. However, 42% HFD for 12 weeks dose not induce any liver changes in Sprague-Dawely rats which were similar as our current study design [41]. Similar to published data showing the beneficial effects of rutin on diet-induced dyslipidemia [21], 12 weeks of rutin supplementation in the present study significantly attenuated HFD-increased serum and hepatic lipid parameters and decreased the atherogenic index. Moreover, total fecal lipids were increased in the HFD + Rutin group, implying that increased total fecal lipid excretion may contribute to a reduction in body weight and serum lipids in the HFD + Rutin group.

Concerning the association between rutin and muscle, studies have shown that rutin improves glucose transport in isolated soleus muscles from rats [42,43]. However, the effect of rutin on mitochondrial changes in skeletal muscle during the progression of obesity has never been elucidated. Obesity is closely associated with mitochondrial dysfunction (*i.e*., decreased oxidation capacity and fatty acid oxidation), reduced expression in a cluster of nuclear genes responsible for oxidative metabolism (PGC-1α), and decreased mitochondrial biogenesis (*i.e*., the generation of new mtDNA and proteins) in skeletal muscle [5,6]. Indeed, HFD-induced obesity reduced the number and size of muscle mitochondria observed by TEM, mtDNA content, and AMPK activation, all of which were improved by rutin supplementation in the present study. Therefore, we suggest that rutin plays a preventive role in obesity-induced mitochondrial changes in skeletal muscle.

Mitochondrial dysfunction, including decreased mitochondrial biogenesis and functional capacity, is associated with several transcriptional regulators and enzyme activities in obese skeletal muscle. As a regulator of mitochondrial biogenesis and function, AMPK promotes mitochondrial biogenesis by direct phosphorylation and interaction with PGC-1α, which up-regulates the synthesis of NRF1 and Tfam. PGC-1α activity is increased by SIRT1-induced deacetylation. AMPK also regulates energy metabolism by increasing the gene expression of PGC-1α and SIRT1, which are involved in fatty acid oxidation [7,8,9,10,44]. All key regulators of muscle mitochondrial biogenesis and function, such as NRF1, Tfam, PGC-1α, and SIRT1, were significantly decreased by HFD and then were fully recovered by rutin supplementation in our current study. NRF1 stimulates the expressions of Tfam, a key nuclear-encoded transcription factor of mtDNA transcription [45,46]. Another mitochondrial enzyme involved in fatty acid oxidation [15], CPT-1, was also significantly increased with rutin. Increased transcription of mtDNA initiates mitochondrial biosynthesis, ultimately leading to mitochondrial abundance in size and number [11,47]. However, rutin-modified mitochondrial size and number were not directly measured in the present study. Using more precise and direct methods such as digital imaging software and stereological principles of point sampling in a blind fashion needs to be taken into consideration to evaluate the role of rutin on muscle mitochondrial size and volume density in the future study [5,48,49]. In addition, molecular mechanisms by which rutin improves mitochondrial function in muscle of obese rats have not been fully determined. Abnormalities in both mitochondrial biogenesis and mitochondrial function including ATP synthesis, oxidative respiration, and intracellular calcium and nitric oxide production are involved in increased reactive oxygen species (ROS) production and endoplasmic reticulum (ER) stress in aging, heart failure, insulin resistance, and obesity [50,51,52,53]. Therefore, further investigation is needed to determine whether rutin affects mitochondrial contents of ATP, calcium, ROS, and ER stress. Given association between SIRT1 and muscle mitochondrial biogenesis and function, actions of rutin-induced quercetin cannot be ruled out. Indeed, rutin supplemented diet significantly increases plasma quercetin concentration [54,55,56], which is involved in the activation of SIRT1, a NAD-dependent deacetlyase [57]. In human fecal microbiota, rutin is involved in bacterial metabolism and action in the gut in relation to fat uptake [58]. In this regard, there is a possibility that rutin-decreased body weight gain in HF-diet fed rats may be associated with direct effect of gut fat uptake and/or indirect influence of rutin-increased blood quercetin on SIRT1 activation. A following study might be necessary to investigate direct and indirect effects of rutin on obesity.

Taken together, the prevention of obesity-induced muscle mitochondrial loss and the improvement of high diet-reduced mitochondrial gene expression in skeletal muscle may demonstrate the favorable effect of rutin on obesity.

## 5. Conclusions

Our study demonstrated that rutin supplementation decreases high-fat diet-induced weight gain and adipose tissue mass, accompanied by increased mtDNA and mitochondrial gene expression involved in mitochondrial biogenesis and function and AMPK activation in skeletal muscle. These results suggested anti-obesity property of rutin may possibly be associated with rutin-mediated muscle mitochondrial changes. Further studies are warranted to delineate more precise mechanisms by which rutin affects muscle fibers, mitochondrial biogenesis, oxidative capacity and function, and its associated health outcomes. To the best our knowledge, this is the first study to suggest that rutin may be partially associated with increased mitochondrial biogenesis and function in muscle of obese rats.
